# HETERONORMATIVE DEFINITIONS OF SEX: IMPLICATIONS FOR LGBT+ WOMEN’S PREVENTIVE HEALTHCARE

**DOI:** 10.1093/geroni/igac059.1919

**Published:** 2022-12-20

**Authors:** Jessica Noblitt, Anne Barrett

**Affiliations:** Florida State University, Tallahassee, Florida, United States; Florida State University, Tallahassee, Florida, United States

## Abstract

The dominant cultural definitions of sex, which is heteronormative, has implications for preventive health screening among LGB+ women. Medical recommendations for women’s screening exclude some same-sex behaviors from this definition, and they center on reproduction – both of which can discourage LGB+ women’s preventive health screening. Qualitative studies have found that sexual minority women, as well as their doctors, are less likely to see sexual health exams as important for sexual minority women’s healthcare because many are not engaging in penile-vaginal intercourse. However, we are aware of no study that has used a large, nationally representative dataset to examine potential differences in health screening by sexual identity. We used data from the National Health Interview Survey (2018; n=1394) to examine differences by sexual identity in having Pap tests and mammograms. We found that sexual minority women were about 40% less likely than heterosexual women to have ever had a Pap test. Moreover, among sexual minority women, lesbian women were about 50% less likely than bisexual women to have ever had one. Sexual minority women also were 22% less likely than heterosexual women to have had a Pap test in the last 12 months. Differences by sexual identity in receiving mammograms were less striking. We found, however, that bisexuals were 25% less likely than lesbians to have ever had a mammogram. In addition, these differences in health screening were more pronounced in younger than older women.

